# Supplementation of Antipsychotic Treatment with the Amino Acid Sarcosine Influences Proton Magnetic Resonance Spectroscopy Parameters in Left Frontal White Matter in Patients with Schizophrenia

**DOI:** 10.3390/nu7105427

**Published:** 2015-10-22

**Authors:** Dominik Strzelecki, Michał Podgórski, Olga Kałużyńska, Oliwia Gawlik-Kotelnicka, Ludomir Stefańczyk, Magdalena Kotlicka-Antczak, Agnieszka Gmitrowicz, Piotr Grzelak

**Affiliations:** 1 Department of Affective and Psychotic Disorders, Medical University of Łódź, Central Clinical Hospital, ul. Pomorska 251, Łódź 92-213, Poland; okaluzynska@gmail.com (O.K.); oliwia.gawlik@umed.lodz.pl (O.G.-K.); magdalena.kotlicka-antczak@umed.lodz.pl (M.K.-A.); 2 Department of Radiology—Diagnostic Imaging, Medical University of Łódź, Łódź 92-213, Poland; chilam@tlen.pl (M.P.); ludomir.stefanczyk@umed.lodz.pl (L.S.); piotr.grzelak@umed.lodz.pl (P.G.); 3 Department of Adolescent Psychiatry, Medical University of Łódź, Łódź 92-213, Poland; agnieszka.gmitrowicz@umed.lodz.pl

**Keywords:** sarcosine, glutamate, white matter, frontal lobe, ^1^H-NMR spectroscopy, schizophrenia

## Abstract

Dysfunction of the glutamatergic system, the main stimulating system in the brain, has a major role in pathogenesis of schizophrenia. The frontal white matter (WM) is partially composed of axons from glutamatergic pyramidal neurons and glia with glutamatergic receptors. The natural amino acid sarcosine, a component of a normal diet, inhibits the glycine type 1 transporter, increasing the glycine level. Thus, it modulates glutamatergic transmission through the glutamatergic ionotropic NMDA (*N*-methyl-d-aspartate) receptor, which requires glycine as a co-agonist. To evaluate the concentrations of brain metabolites (NAA, *N*-acetylaspartate; Glx, complex of glutamate, glutamine, and γ-aminobutyric acid (GABA); mI, *myo-*inositol; Cr, creatine; Cho, choline) in the left frontal WM, Proton Nuclear Magnetic Resonance (^1^H-NMR) spectroscopy was used. Twenty-five patients randomly chosen from a group of fifty with stable schizophrenia (DSM-IV-TR) and dominant negative symptoms, who were receiving antipsychotic therapy, were administered 2 g of sarcosine daily for six months. The remaining 25 patients received placebo. Assignment was double blinded. ^1^H-NMR spectroscopy (1.5 T) was performed twice: before and after the intervention. NAA, Glx and mI were evaluated as Cr and Cho ratios. All patients were also assessed twice with the Positive and Negative Syndrome Scale (PANSS). Results were compared between groups and in two time points in each group. The sarcosine group demonstrated a significant decrease in WM Glx/Cr and Glx/Cho ratios compared to controls after six months of therapy. In the experimental group, the final NAA/Cr ratio significantly increased and Glx/Cr ratio significantly decreased compared to baseline values. Improvement in the PANSS scores was significant only in the sarcosine group. In patients with schizophrenia, sarcosine augmentation can reverse the negative effect of glutamatergic system overstimulation, with a simultaneous beneficial increase of NAA/Cr ratio in the WM of the left frontal lobe. Our results further support the glutamatergic hypothesis of schizophrenia.

## 1. Introduction

The role of the white matter (WM) in the pathogenesis of schizophrenia is underestimated and relatively poorly explored, compared with other brain regions, perhaps as a consequence of attributing the etiology of schizophrenia chiefly to grey matter dysfunctions. The approach resembles an evolving concept of the hierarchy of transmitter systems playing a key role in pathogenesis of schizophrenia [1,2,3,4,5]. It used to be believed that dopaminergic system impairment was responsible for the development of schizophrenia, followed later by the serotoninergic system disturbances and dysfunction of stimulatory/inhibitory role of the glutamatergic and GABAergic (GABA, γ-aminobutyric acid) systems [6]. The role of the latter was confirmed by the fact that administration of phencyclidine or ketamine, a glutamatergic NMDA (*N*-methyl-d-aspartate) receptor antagonist, could cause psychotic states, mimicking schizophrenia or aggravating symptoms in patients with schizophrenia [7,8,9]. Furthermore, this effect is mostly noticeable after reaching adolescence [10], co-occurring with the peak of schizophrenia, which is consistent with the neurodevelopmental theory.

Dopamine receptor antagonists still constitute a first line intervention in the management of schizophrenia. These medications are effective in treating positive symptoms (delusions, hallucinations, behavior, and thought disorders). However, standard antipsychotic therapy does not usually sufficiently improve other groups of symptoms that have a major impact on general prognosis and quality of life: negative symptoms (anhedonia and avolition, autistic behavior, emotional flattening, social withdrawal); affective symptoms (sadness, anxiety); or cognitive symptoms (disturbances of working memory, attention, and executive functions). Thus, standard antipsychotic therapy results in low rates of recovery, typically around 13.5% [11].

One experimental therapy intended for treating negative and cognitive symptoms aims to modulate ionotropic glutamatergic NMDA receptor function. It can be achieved by supplementation of natural NMDA receptor co-agonist (glycine), substances having similar properties (d-serine, d-cycloserine), or inhibitor of glycine transporter type 1 (GlyT1) (sarcosine, bitopertin) [12,13,14,15,16,17].

Sarcosine is a component of a conventional diet, and can be found in eggs, vegetables (e.g., legumes, nuts, roots), poultry, and other meats. In small concentrations, sarcosine is present in almost all biological materials and in human tissues including the muscles, liver, and kidneys. Sarcosine is present in the blood serum of subjects not receiving supplementation, at mean concentrations of 102.3 ng/mL in men and 80.8 ng/mL in women [18]. Sarcosine is also formed from dietary choline and from methionine metabolism, and is degraded to glycine by sarcosine dehydrogenase, while glycine-*N*-methyl transferase generates sarcosine from glycine [19]. Sarcosine is sweet to the taste and dissolves in water. It is widely used in cosmetic formulations (toothpastes, cremes, soaps) or in the manufacture of biodegradable surfactants. Supplementation with 2 g of sarcosine daily may reduce the hypofunction of the NMDA receptor on GABAergic inhibiting interneurons [20,21,22]. Hence, enhancement of dysfunctional inhibition may reduce information overload, preventing or reversing the occurrence of positive symptoms, cognitive impairment and improving weak social functioning [22]. NMDA receptors are present in high densities in the prefrontal cortex (PFC) and hippocampus [23,24,25]. These regions are fundamental in the pathogenesis of schizophrenia and their axons contribute to the WM of the frontal lobes. Furthermore, glutamatergic receptors, including NMDA receptors, are present on glial cells that support and modify the function of the aforementioned WM [5,26,27]. In addition, numerous studies indicate that schizophrenia susceptibility genes are involved in glutamatergic transmission, suggesting that the glutamatergic transmission system plays a role in the neurodevelopmental hypothesis of schizophrenia [28,29].

Recently, glutamatergic receptors (e.g., NMDA and AMPA, α-amino-3-hydroxy-5-methyl-4-isoxazolepropionic acid) have been found in glial cells in the white matter. These receptors might have an excitotoxic effect on neurons, becoming a target for therapeutic agents [30]. Magnetic resonance imaging has revealed many disturbances in the function and density of glial cells, particularly the oligodendrocytes forming myelin, in the prefrontal WM of patients with schizophrenia [31,32,33,34]: decreased regional and global WM volumes [35,36,37,38], decreased ratios of magnetization transfer [39,40] and anisotropy of diffusion tensor imaging [41,42,43,44,45], all of which may indicate a possible pathology of myelin and WM in general [46,47].

The proton nuclear magnetic resonance spectroscopy (^1^H-MRS) technique used in the present study allows non-invasive, *in vivo* evaluation of metabolite concentrations. Other studies based on this technique have found the most common differences between patients with schizophrenia and healthy volunteers to be overall decreased *N*-acetylaspartate (NAA) and *myo*-inositol (mI) levels [46]. In addition, Glx (a complex of glutamate, glutamine and, partially, GABA) or glutamate concentrations are noticed as increased, stable or decreased [46]. Choline (Cho) and creatine (Cr) levels are stable [48]. *N*-acetylaspartate is a marker of neuronal viability and integrity with its highest concentrations in pyramidal glutamatergic neurons [49,50]. Thus, decreased NAA concentration in WM suggests decreased neuronal content and/or neuronal dysfunction [46,51,52].

*Myo*-inositol is a glial marker. Hence, its decreased concentration observed in the WM of patients with schizophrenia suggests that the density of neuroglia is decreased or WM is not functioning correctly [46].

Spectroscopic examinations performed with a 1.5 T (Tesla) magnetic field reveal peaks corresponding to glutamate (Glu), glutamine (Gln), and GABA overlapping each other. However, as Glu concentration is five times higher than the concentration of Gln, and ten times higher than GABA, Glx, Glx/Cr, and Glx/Cho are accepted as markers of glutamatergic transmission [53]. Moreover, Glu and Gln are closely-associated metabolites. When Glu is released from neurons, it is up-taken by glia and converted to Gln, which is further transferred to neurons and converted again to Glu [54,55]. Further spectroscopic studies also reveal dysfunctions in glutamatergic transmission in various brain areas. Unmedicated patients demonstrated increased levels of Glx and GABA in the medial PFC, but not the dorso-lateral PFC, compared to those of patients receiving antipsychotic medication [56]. An increased Glx/Cr ratio was also found in the hippocampal complex in schizophrenia [57]. A similar increase of glutamate levels was found in the precommissural dorsal-caudate of ultra-high risk and first-episode patients; however, no differences were noted in parameters of the cerebellum in these groups [58]. Elevated levels of Glx in the WM in schizophrenia may reflect a hyperglutamatergic state, leading to information redundancy and excitotoxicity [46].

Measuring the ratio to Cho and Cr is an accepted method of evaluating brain metabolite concentrations. Despite the changes in Cho and Cr concentrations occurring with schizophrenia progression, previous studies suggest that treatment with either atypical or typical medication does not alter Cr or Cho levels [55]. A large meta-analysis of ^1^H-MRS studies in schizophrenia showed no significant differences in either Cho or Cr in WM [59]. Hence, this ratio might have good intra-subject validity [55].

Existing spectroscopy-based studies do not provide consistent conclusions on metabolite concentrations in schizophrenia, especially when considering different brain regions, aggravation of symptoms, different phases of the disease or treatment strategy [48,59,60,61]. The aim of the present study is to evaluate the influence of six months of sarcosine therapy on the concentration of NAA, Glx, mI, Cho, and Cr in the left frontal WM in patients with stable schizophrenia.

## 2. Experimental Section

### 2.1. Materials and Methods

The following inclusion criteria applied for entry to the study: age 18–60 years, in good physical, neurological, and endocrinological health, with laboratory test results within normal values (routine blood tests, biochemical tests including TSH (thyroid-stimulating hormone), lipid profile, liver and kidney parameters, and ECG (electrocardiogram). Patients in acute psychosis, on clozapine treatment or declaring suicidal tendencies were excluded from the study. Sources for further details regarding the Polish Sarcosine Study in Schizophrenia (PULSAR) are given in the acknowledgments.

Fifty right-handed patients in a stable clinical condition who had been diagnosed with schizophrenia (according to DSM-IV-TR criteria), with dominant negative symptoms were selected from admissions to the outpatient clinic. They were randomly assigned in a double-blinded manner to a sarcosine or a placebo group using the protocol obtained from a dedicated website [62]. In both groups, matched according to age, sex, clinical presentation treatment, duration of schizophrenia and education (Table 1), ^1^H-NMR spectroscopy was performed at the beginning of the study and after six months according to the protocol described below.

**Table 1:** Characteristics of groups.

Features		Group		*p*-value
		Sarcosine (*n* = 25)	Control (*n* = 25)	
Gender	Female	14	12	>0.05
	Male	11	13	
Age (mean, year)		36.5	40	>0.05
Mean number of hospitalizations		5	4	>0.05
Mean duration of the illness (years)		12.3	13.2	>0.05
Mean timespan of education per patient (years)		14.2	14.4	>0.05
Smoking		9	11	>0.05
Antipsychotic treatment (DDD)		1.94	1.97	>0.05
Anti-depressive treatment (DDD)		0.58	0.6	>0.05
PANSS total (± SD)		71.4 ± 14	73.3 ± 13	>0.05

Abbreviations: DDD: defined daily dose; PANSS: the Positive and Negative Syndrome Scale, total score; SD: standard deviation.

Sarcosine and placebo were added to the ongoing antipsychotic treatment. Patients in the study group were given plastic capsules containing 2 g of the amino acid, while subjects in the control group received capsules with microcrystalline cellulose. Both groups of subjects were asked to drink the dissolved contents of one capsule once a day in the morning. The patients were asked to bring all capsules, including empty ones, to each visit; these were compared with the number of days since the last visit. Derogations were discussed with the patients. All patients had been treated with stable doses of antipsychotic medication (excluding clozapine) for a minimum period of three months before baseline visit. The doses of antipsychotic and anti-depressive drugs were calculated for defined daily dose (DDD) developed by the World Health Organization. Antidepressants were used as a typical supportive therapy for affective and negative symptoms [12]. While antidepressive drugs were administered to 14 patients in the sarcosine group but only 11 in the control group, this difference was not significant (*p* = 0.43211).

The severity of schizophrenia symptoms was assessed with the Positive and Negative Syndrome Scale (PANSS) [63]. According to the results of the Chi^2^ test, no significant difference existed between groups with regard to the number of smokers. In addition, the spectroscopic examinations were performed in a short period of time, and the smoking status was not observed to change in any subject.

Subjects were recruited from an outpatient clinic. All patients included in the study were informed about the aims and methods of the study and gave their written informed consent for participation. The Bioethics Committee of the Medical University of Łódź approved the study protocol (permission number and date: RNN/153/08/KE, 15 July 2008). The study received no commercial financial involvement.

### 2.2. Spectroscopy

Imaging was performed using a 1.5 T magnetic resonance scanner (Siemens Avanto 1.5, Munich, Germany) equipped with a standard head NMR (nuclear magnetic resonance) acquisition coil:

1. FLAIR (fluid-attenuated inversion recovery) sequences in axial plane with following parameters: Repetition Time (TR), 9000 ms; Echo Time (TE), 105 ms; inversion time (TI), 2500 ms; flip angle, 150°; voxel size 1.4 × 1.3 × 3 mm.
2. T2-weighted sequences were obtained in coronal plane with following parameters: TR = 5000 ms; TE = 100 ms; flip angle, 50°; voxel size 0.6 × 0.6 × 5.0 mm.
3. T1 weighted sequences in transverse plane with following parameters: TR = 400 ms; TE = 7.8 ms; flip angle, 90° g; voxel size 0.9 × 0.9 × 0.5 mm.

**Figure 1:**
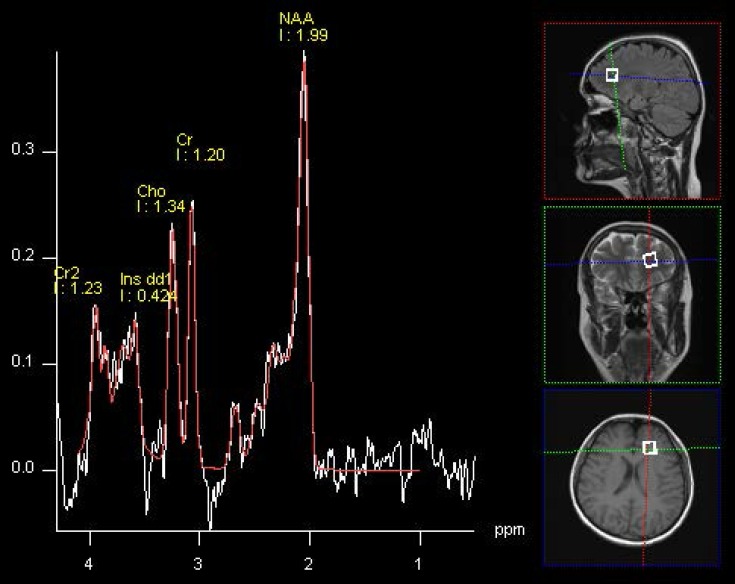
Images showing voxel location in the left WM area and an example of spectra before (white line) and after (red line) fitting. Peak areas for *N*-acetylaspartate (NAA); creatine (Cr and Cr2); choline (Cho); and *myo*-inositol (Ins dd1) are labeled.

^1^H-MRS data was acquired by single voxel spectroscopy (SVS) using a point resolved spin echo (PRESS) sequence 128 averages; TR, 3000 ms; TE, 30 ms; voxel size was 15 × 15 × 15 mm. The region of interest was placed in a left frontal WM by the neuroradiologist (Figure 1). During the second spectroscopic examination, the voxel localization parameters were copied and adjusted to the position of the patient to evaluate the same region. Automated procedures were used to optimize radio-frequency pulse power, field homogeneity, and water suppression, as well as to convert the lines into a Gaussian shape. Water suppression was achieved by a pulse transmitted by the scanner at the beginning of the spectroscopic sequence. Spectroscopy data was processed with Avanto Syngo MR Software (Siemens, Munich, Germany), Level B15, using the following protocols: k-space Fourier transformation and a spatial 50 Hz Hanning filter; subtraction of the residual water signal; time domain 1 Hz exponential apodization; zero filling to 2048 points; Fourier transformation of the time domain signals; frequency shift correction, phase correction, and baseline correction. The fitting error was automatically calculated as a deviation between the theoretical and measured spectra determined using the last squares method. Values less than 0.5 were considered satisfactory; however, mean fitting error was 0.33 in the whole group (standard deviation, SD = 0.03). Ratios of metabolite concentrations to Cr and Cho were determined instead of absolute concentrations.

### 2.3. Statistical Analysis

Continuous variables are expressed as the mean ± standard deviation (SD). The Shapiro-Wilk test was used to determine the normality of the data. As they were found to have a skewed distribution, the Mann–Whitney test was used to compare of ratios of substance concentrations between groups, and the Wilcoxon sign rank test was used to identify relationships within the same group. As concentrations of brain metabolites are affected by age and smoking status, these parameters were used as covariates in multiple stepwise regression analysis, together with group affiliation. The change in metabolite concentration ratio for each metabolite was calculated and used as a dependent variable. Statistical analysis was performed using Statistica for Windows (version 12.0, StatSoft, Tulsa, OK, USA). A *p*-value of ≤0.05 was considered significant.

## 3. Results

At baseline, no differences were found between the two groups regarding spectroscopic parameters (Table 2).

**Table 2:** Comparison of substance concentration ratios at the beginning and the end of the study.

Compared Ratios	Baseline			After 6 months			Baseline *vs.* after 6 months	Baseline *vs.* after 6 months
	Sarcosine (mean ± SD)	Placebo (mean ± SD)	*p*-value	Sarcosine (mean ± SD)	Placebo (mean ± SD)	*p*-value	Sarcosine *p*-value	Placebo *p*-value
NAA/Cr	1.96 (2.55)	2.43 (1.79)	>0.05	2.37 (0.53)	2.75 (1.37)	>0.05	0.0048	>0.05
Cho/Cr	1.31 (0.87)	1.21 (0.84)	>0.05	1.02 (0.34)	1.05 (0.86)	>0.05	>0.05	>0.05
mI/Cr	0.28 (0.18)	0.37 (0.28)	>0.05	0.28 (0.15)	0.30 (0.14)	>0.05	>0.05	>0.05
Glx/Cr	0.73 (0.33)	0.75 (0.20)	>0.05	0.51 (0.21)	0.80 (0.20)	0.0062	0.0281	>0.05
NAA/Cho	2.34 (0.84)	2.18 (0.83)	>0.05	2.30 (1.72)	2.65 (2.40)	>0.05	>0.05	>0.05
mI/Cho	0.27 (0.25)	0.30 (0.23)	>0.05	0.27 (0.15)	0.31 (0.13)	>0.05	>0.05	>0.05
Glx/Cho	0.75 (0.27)	0.83 (0.20)	>0.05	0.57 (0.18)	0.85 (0.20)	0.0041	>0.05	>0.05

Abbreviations: NAA: *N*-acetylaspartate; Cr: creatine; Cho: choline; mI: *myo*-inositol; Glx: glutamate glutamine, and GABA; SD: standard deviation.

At the second assessment, Glx/Cr and Glx/Cho ratios were significantly decreased in the sarcosine group compared to controls: 30% and 24% decrease in the sarcosine group *vs.* 6.6% and 2.4% increase in the control group, respectively (Table 2). Following therapy, NAA/Cr significantly increased by 21% (*vs.* 13% in controls) and Glx/Cr ratios significantly decreased (as above) in the experimental group compared to baseline values (Table 2).

According to the regression analysis (Table 3) changes in NAA/Cr ratios were due to age and smoking status differences between groups. Age has also a significant effect on mI/Cho ratio change. However, the Glx/Cr ratio change was due to administration of sarcosine. Furthermore, influence of sarcosine on Glx metabolism was also suggested by significant change in Glx/Cho ratio due to its supplementation.

**Table 3:** Multiple stepwise regression analysis of the determinants of substance concentration ratios in the left frontal WM. The table only contains data for metabolite concentrations that were predicted by the regression model.

Difference in Concentration Ratio	Predictor	β—Coefficient (± SD)	Corrected *R*^2^ of the Model	*p* Value
NAA/Cr	Age	−0.0234 (0.0097)	0.1565	0.0208 *
	Smoking	0.3629 (0.1978)		0.0733
Glx/Cr	Group	0.2079 (0.0616)	0.1844	0.0015 *
NAA/Cho	Age	0.02679 (0.0194)	0.0192	0.1748
mI/Cho	Age	0.0038 (0.0018)	0.0705	0.0396 *
Glx/Cho	Group	0.12943 (0.0562)	0.1207	0.0260 *
	Smoking	−0.09086 (0.0565)		0.1150

Group: group affiliation; Smoking: smoking status; *: statistically significant; SD: standard deviation.

At the beginning of the experiment, no significant differences in PANSS scores were found between groups: 71.4 ± 14 (sarcosine) *vs.* 73.3 ± 13 (placebo) (*p* = 0.6736). However, at the end of the experiment, patients treated with sarcosine had significantly lower results: 57.7 ± 15 (sarcosine) *vs.* 71.5 ± 13 points (placebo) (*p* = 0.00487).

## 4. Discussion

To our knowledge, this is the first study to evaluate the effect of a glutamatergic modulator (GlyT1 inhibitor) on ^1^H-NMR spectroscopy parameters in the white matter of patients with schizophrenia. Differences in NAA/Cr, Glx/Cr and Glx/Cho ratios were observed between sequential examinations in the experimental group, and between both groups after therapy. The findings suggest that a daily dose of 2 g of sarcosine may cross the blood-brain barrier, influence glutamatergic transmission, and improve the mental state of schizophrenia patients.

Morphological and functional abnormalities of various regions of the brain are known to be involved in the pathogenesis of schizophrenia, along with disturbed connectivity between the regions. The WM is responsible for functional connectivity in the brain, and consists mainly of neuronal axons and glia (particularly for the sake of this paper, astrocytes and oligodendrocytes. Oligodendrocytes provide required insulation of neuronal fibers; their membranes surround axons with myelin sheaths. Astrocytes, besides providing structural and metabolic functions, are involved in transmitter re-uptake and release, modulation of synaptic transmission, vasomodulation, promotion of the myelinating activity of oligodendrocytes, and neural tissue repair processes [64]. Axonal, astroglial, and oligodendroglial pathologies are established in schizophrenic process and they will be shortly described to aid further discussion.

### 4.1. Axonal Pathology

Diffusion tensor imaging (DTI), a magnetic resonance technique, is a noninvasive method for the evaluation of axonal tract pathways and their structure. This technique enables the quantity and integrity of axonal tracts to be the evaluated by fractional anisotropy (FA); axial diffusivity (AD)—diffusion in the principal direction of fascicles, indicating the degeneration of axons; and radial diffusivity (RD)—diffusion perpendicular to the direction of fascicles, indicating the degeneration of myelin [65,66]. Conclusions from DTI studies indicate differences in fiber orientation, axonal packing density, membrane permeability, and increased dysmyelination in schizophrenia, comparing to healthy populations [67,68,69]. Furthermore, in the chronic phase of schizophrenia, FA was significantly decreased in multiple regions of the WM throughout the brain compared with controls [70,71,72,73,74]. Although the significance of the changes in first-episode patients was only thought to be a trend, longitudinal studies indicate that these changes have a progressive character [75,76].

Progression was also observed in a high-resolution multispectral magnetic resonance study: A gradual reduction in volume of the frontal WM was noted during three years of observation [77].

### 4.2. Astroglia Pathology

Increased numbers of astrocytes are commonly observed in patients with schizophrenia (astrogliosis), which is generally typical of degenerative processes of the central nervous system. Astroglia play a major role in neurotransmission by forming the tripartite synapse with neurons, which is responsible for the release and control of neurotransmitter levels [78,79]. However, although the number of glia is increased in astrogliosis, neuronal and glial dysfunctions prevent them from functioning correctly.

### 4.3. Olidendroglia Pathology

Several studies have suggested the presence of oligodendroglial dysfunction in schizophrenia, occurring as a result of atypical myelin maintenance and repair in the deeper regions of the WM [80,81]. Oligodendroglial abnormalities observed in schizophrenia are greater dispersion and decreased density of glia [33,34], reduced population [34,82,83] and aberrant morphology (damage of myelin sheaths, necrotic and apoptotic changes) [66,82,84]. These changes have also been associated with changes of genes related to oligodendrocytes and myelin [85,86].

### 4.4. Analysis of Spectroscopic Parameters

#### 4.4.1. Glx

It is postulated that in schizophrenia, glutamatergic system hyperactivity results from impaired control of GABAergic interneurons, and disturbed interactions with the dopaminergic system [22,87,88]. Spectroscopic studies commonly find increased levels of Glx or Glx/Cho and Glx/Cr ratios in the WM, indicating the presence of glutamatergic overstimulation. As these changes are associated not only with the degeneration of axonal fibers but also with disequilibrium between the stimulation of glutamatergic transmission and inhibition of GABAergic transmission, they are thought to be markers of altered neuron activity, function, and structure.

On the other hand, insufficient glutamate re-uptake can lead to increased glutamate toxicity, which in turn can induce glutamatergic transmission hyperactivity and astroglia dysfunction. The myelin and oligodendroglia can also be affected, as these are particularly susceptible to glutamatergic excitotoxic damage [89,90].

This is the first report to indicate that increased glutamatergic activity in the WM can be pharmacologically reversed in schizophrenia, with accompanying clinical improvement. Six months of therapy with sarcosine can, therefore, positively affect glutamatergic transmission, as expressed by a significant decrease of Glx/Cr and Glx/Cho ratios in patients with schizophrenia compared with controls, as well as by a decreased Glx/Cr ratio revealed by a follow-up examination in the experimental group. The fact that the total PANSS score decreased by 19.2% in the experimental group, with no significant changes in the control group, indicates that the spectroscopic changes observed in patients with schizophrenia were favorable. Moreover, it may indicate an association between glutamatergic transmission in the WM and symptoms of schizophrenia.

#### 4.4.2. NAA

Although the precise role of NAA in central nervous system is uncertain, it is probably engaged in the metabolism of other amino acids, such as glutamate. It is specifically synthesized in high concentrations in the neuronal mitochondria, but not in glia, and its production is closely linked with the neuronal glucose metabolism [91]. Hence, NAA is believed to be a spectroscopic marker of neuronal vitality.

Neuropathological studies in schizophrenia have found a reduction of brain volume [92], neuronal soma size [93], and dendritic abnormalities [94,95]. In addition, although the density of the neuronal network might be greater, the neurons within it can be incorrectly located and connected [96,97]. These features of the WM and the changes found in DTI in patients with schizophrenia account for the possible permanent decreases observed in NAA levels or NAA/Cr and NAA/Cho ratios.

In the present study, the voxel was positioned in close proximity to the PFC (dorsolateral and dorsomedial), whose dysfunction in schizophrenia is associated with negative symptoms and cognitive function impairment [98]. These groups of symptoms are especially resistant to contemporarily-recommended therapies and are the major cause of impaired general functioning of patients with schizophrenia.

The observed significant increase of NAA/Cr ratio in patients receiving sarcosine, together with the simultaneous improvement of their clinical condition, suggests the presence of increased neuronal activity within the frontal WM. Moreover, it can also be an indicator of the better condition of the neurons of the PFC, whose axons contribute to the examined region of the WM. This observation is important from the clinical point of view because cognitive impairment level is known to be the best predictor of long-term functional outcome in patients with schizophrenia: better than the severity of positive, negative or affective symptoms [99].

#### 4.4.3. mI

Myoinositol, a precursor in the phosphatidylinositol signaling system, is also widely accepted as a glial marker in spectroscopic studies [100]. Generally, in neurodegenerative processes, mI levels fell, together with NAA levels, as dementia progressed [101].

Myoinositol ratios remained relatively stable, both within each group and between groups. This stability may indicate that sarcosine-induced changes in brain metabolism did not significantly affect glia cells.

### 4.5. Limitations of the Study

This research has a few limitations. It was performed with the application of a 1.5 T magnetic field: A stronger magnetic field (e.g., 3 T) would allow overlapping peaks of glutamate, glutamine, and GABA to be separated. It would be particularly interesting to assess GABA concentration, because of the indirect action of sarcosine on NMDA receptors located on GABAergic interneurons.

Phantom studies were not performed, even though these would allow exact concentrations to be evaluated instead of ratios. Moreover, although treatment with antipsychotic does not alter Cr or Cho levels [55], there is no data on the effect of sarcosine on their concentrations. Nevertheless, Cho is a marker of cell membrane turnover, which is elevated in neoplasms, demyelination, and gliosis, and Cr tends to be maintained at a relatively constant level, as it is associated with the storage and transfer of energy [102]. In addition, the intra-subject validity of ratios was reported to be adequate [55]. Hence, sarcosine should not influence Cho and Cr concentrations and, despite the limitations given above, our conclusions seem to be reliable.

Another limitation of this study concerns the lack of determination of the exact amount of WM and gray matter encompassed within the voxel volume. However, due to the fact that a small voxel size was used and that patients were young (the mean age in experimental and control groups were 36.5 and 40 years respectively), the amount of WM in the region of interest was sufficient to contain the whole voxel volume.

Finally, the applied statistical methods may increase the risk of a type II error associated with the application of a non-parametric test instead of a parametricone, and a type I error caused by the multiple testing of different ratios between groups. However, the clinical improvement observed in the patients and the medical relevance of the observed changes seems to confirm the reliability of the obtained results.

## 5. Conclusions

This is the first report to indicate that in patients with stable schizophrenia, the addition of 2 g of sarcosine to standard antipsychotic therapy for six months may reverse the negative influence of glutamatergic transmission system dysfunctions with a simultaneous improvement of neuronal vitality markers in the WM of the left frontal lobe. Moreover, the presented results confirm that the glutamatergic system is involved in the pathogenesis of schizophrenia, and that the GlyT1 inhibitor may positively affect the metabolism of the WM and schizophrenia symptoms.
